# Survival of ship biofouling assemblages during and after voyages to the Canadian Arctic

**DOI:** 10.1007/s00227-016-3029-1

**Published:** 2016-11-11

**Authors:** Farrah T. Chan, Hugh J. MacIsaac, Sarah A. Bailey

**Affiliations:** 1 Great Lakes Institute for Environmental Research, University of Windsor, Windsor, ON N9B 3P4 Canada; 2 Great Lakes Laboratory for Fisheries and Aquatic Sciences, Fisheries and Oceans Canada, Burlington, ON L7S 1A1 Canada

## Abstract

Human-mediated vectors often inadvertently translocate species assemblages to new environments. Examining the dynamics of entrained species assemblages during transport can provide insights into the introduction risk associated with these vectors. Ship biofouling is a major transport vector of nonindigenous species in coastal ecosystems globally, yet its magnitude in the Arctic is poorly understood. To determine whether biofouling organisms on ships can survive passages in Arctic waters, we examined how biofouling assemblage structure changed before, during, and after eight round-trip military voyages from temperate to Arctic ports in Canada. Species richness first decreased (~70% loss) and then recovered (~27% loss compared to the original assemblages), as ships travelled to and from the Arctic, respectively, whereas total abundance typically declined over time (~55% total loss). Biofouling community structure differed significantly before and during Arctic transits as well as between those sampled during and after voyages. Assemblage structure varied across different parts of the hull; however, temporal changes were independent of hull location, suggesting that niche areas did not provide protection for biofouling organisms against adverse conditions in the Arctic. Biofouling algae appear to be more tolerant of transport conditions during Arctic voyages than are mobile, sessile, and sedentary invertebrates. Our results suggest that biofouling assemblages on ships generally have poor survivorship during Arctic voyages. Nonetheless, some potential for transporting nonindigenous species to the Arctic via ship biofouling remains, as at least six taxa new to the Canadian Arctic, including a nonindigenous cirripede, appeared to have survived transits from temperate to Arctic ports.

**Electronic supplementary material:**

The online version of this article (doi:10.1007/s00227-016-3029-1) contains supplementary material, which is available to authorized users.

## Introduction

The introduction of nonindigenous species (NIS) by human activities is a key component of global environmental change (Simberloff et al. 2013). While many NIS have negligible or even beneficial effects, a subset is detrimental to recipient environments by causing extinctions of native species, modifying habitat structure, and disrupting ecosystem functions and services, among other effects (Blackburn et al. 2014; Gallardo et al. 2016). To successfully establish, NIS must survive uptake and transport by a vector before they can be introduced and form persisting populations at new locations, provided that the biotic and abiotic conditions are suitable (Blackburn et al. 2011). Many marine vectors, such as ships’ ballast water and fouled hulls, marine debris, and bait worm packaging, often inadvertently translocate large, mixed-species assemblages during a single introduction event (e.g. Gregory 2009; Sylvester et al. 2011; Briski et al. 2013; Fowler et al. 2016). In general, only a fraction of individuals and species survive transport and are introduced to a new location (Lockwood et al. 2009; Briski et al. 2014; Chan et al. 2015a). This is because conditions during the transport process are usually hostile; for instance, physical disturbance and fluctuations in environmental conditions during transport may selectively affect survivorship of entrained organisms (Coutts et al. 2010a; Clarke Murray et al. 2012; Briski et al. 2014). Therefore, examining the fate of organisms and how assemblage structure varies during transport can provide insights into the introduction potential associated with multispecies vectors (Briski et al. 2014; Chan et al. 2015a).

Ship biofouling, defined as the accumulation of organisms on exterior wetted ship surfaces, is a leading transport vector of NIS in coastal ecosystems globally (e.g. Hewitt et al. 2009; Williams et al. 2013). A wide range of mobile, sessile, and sedentary organisms colonize hull surfaces and can dislodge and/or reproduce at subsequent ports-of-call (Apte et al. 2000; Minchin and Gollasch 2003; Chapman et al. 2013). Given the propensity of ships to transport NIS on their hulls, considerable research has been undertaken to characterize the composition of biofouling assemblages and identify factors that may influence fouling extent on ships in temperate waters (e.g. Coutts et al. 2010a; Sylvester et al. 2011; Clarke Murray et al. 2012). Very few studies, however, have examined pre- and post-voyage survivorship of biofouling organisms on ships by repeated sampling (Carlton and Hodder 1995; Brock et al. 1999; Davidson et al. 2008; Coutts et al. 2010a, b). The majority of these studies were conducted on experimental plates (Coutts et al. 2010b) or on slow-moving, obsolete ships (Carlton and Hodder 1995; Brock et al. 1999; Davidson et al. 2008). Only Coutts et al. 2010a quantified en route survivorship of biofouling taxa on operating ships, but during short trips (<12 h).

The importance of ship biofouling as a vector for delivery of NIS to the Arctic is unclear. To date there has been only one post-voyage in situ assessment of biofouling on commercial ships conducted in the Canadian Arctic, the results of which represent a snapshot of biofouling at one site (Chan et al. 2015b). In contrast, ship biofouling has received far more attention in the Antarctic, where survivorship of biofouling organisms during voyages from temperate to Antarctic and sub-Antarctic ports has been characterized (Lewis et al. 2004; Lee and Chown 2009; Hughes and Ashton 2016). In general, passage through sea ice effectively removed biofouling assemblages attached to the hull of ships travelling to Antarctic ports (Lewis et al. 2004; Lee and Chown 2009), but organisms in niche areas such as intake ports and sea chests were protected from ice and had higher survival during transits (Hughes and Ashton 2016). Furthermore, a large proportion of the initial biofouling assemblages typically remained on ships’ hulls following ice-free voyages to sub-Antarctic ports (Lewis et al. 2004; Lee and Chown 2009). These results have raised concerns about the potential for transferring temperate marine species to polar regions via ship biofouling (Lewis et al. 2004; Lee and Chown 2009; Hughes and Ashton 2016).

Arctic coastal environments are under unprecedented threats from NIS because of climate change, resource development, and expanded Arctic shipping (Miller and Ruiz 2014; Ware et al. 2014, 2016). Rising sea surface temperature in the Arctic over the past three decades has resulted in retreating sea ice (Hoegh-Guldberg and Bruno 2010) and opening of waterways and shipping channels such as the Northern Sea Route and the Northwest Passage (Smith and Stephenson 2013; Pizzolato et al. 2014; Miller and Ruiz 2014). Arctic shipping traffic has increased rapidly as a result of growth in exploration, extraction, and export of natural resources, fisheries, and tourism (Miller and Ruiz 2014). The shipping season has also been extended in some parts of the Arctic because of later ice formation (Pizzolato et al. 2014). New routes, increased shipping intensity, and a prolonged season will increase the diversity and abundance of NIS arriving to the Arctic via shipping (Miller and Ruiz 2014). Once released into the recipient environment, NIS may be able to overcome historic environmental constraints and form persisting populations in the Arctic under a milder climate (Hellmann et al. 2008). Present climatic conditions in some high-latitude systems are already suitable for temperate NIS; thus, successful establishment may be possible given sufficient introduction effort (de Rivera et al. 2011). Further changes in climatic conditions will increase the environmental similarity between temperate and Arctic habitats, thereby increasing the susceptibility of Arctic ecosystems to NIS invasions (Ware et al. 2014). Reduced ice coverage in the Arctic is also expected to enhance coastal productivity, enhancing food supply to suspension-feeding organisms (Vermeij and Roopnarine 2008). Therefore, an examination of introduction risk associated with ship biofouling including an evaluation of survivorship of biofouling assemblages during transits is clearly opportune for the Arctic region.

As mentioned, studies examining the dynamics of entrained assemblages by repeated sampling during transport are typically rare owing to logistic challenges (Briski et al. 2014). We had opportunity to conduct time-point sampling of biofouling assemblages on hulls of several military ships before, during (when possible), and after eight round-trip voyages from temperate (Halifax, Nova Scotia) to Arctic (Iqaluit, Nanisivik, or Resolute in Nunavut or Churchill in Manitoba) ports in Canada. To determine whether biofouling organisms can survive passage through Arctic waters, we examined how biofouling assemblage structure varied over time during these voyages. We hypothesized that biofouling assemblage structure will differ across the three sampling time points (i.e. before, during, and after Arctic voyages) owing to environmental variations. In addition, we tested whether location on the hull has an effect on biofouling assemblage structure (see Davidson et al. 2009; Coutts et al. 2010b; Sylvester et al. 2011) and whether temporal changes in assemblage structure during Arctic voyages depend on hull location. Finally, we explored whether responses of assemblage structure differ across taxonomic groups based on their motility.

## Materials and methods

### Study site

**Fig. 1:**
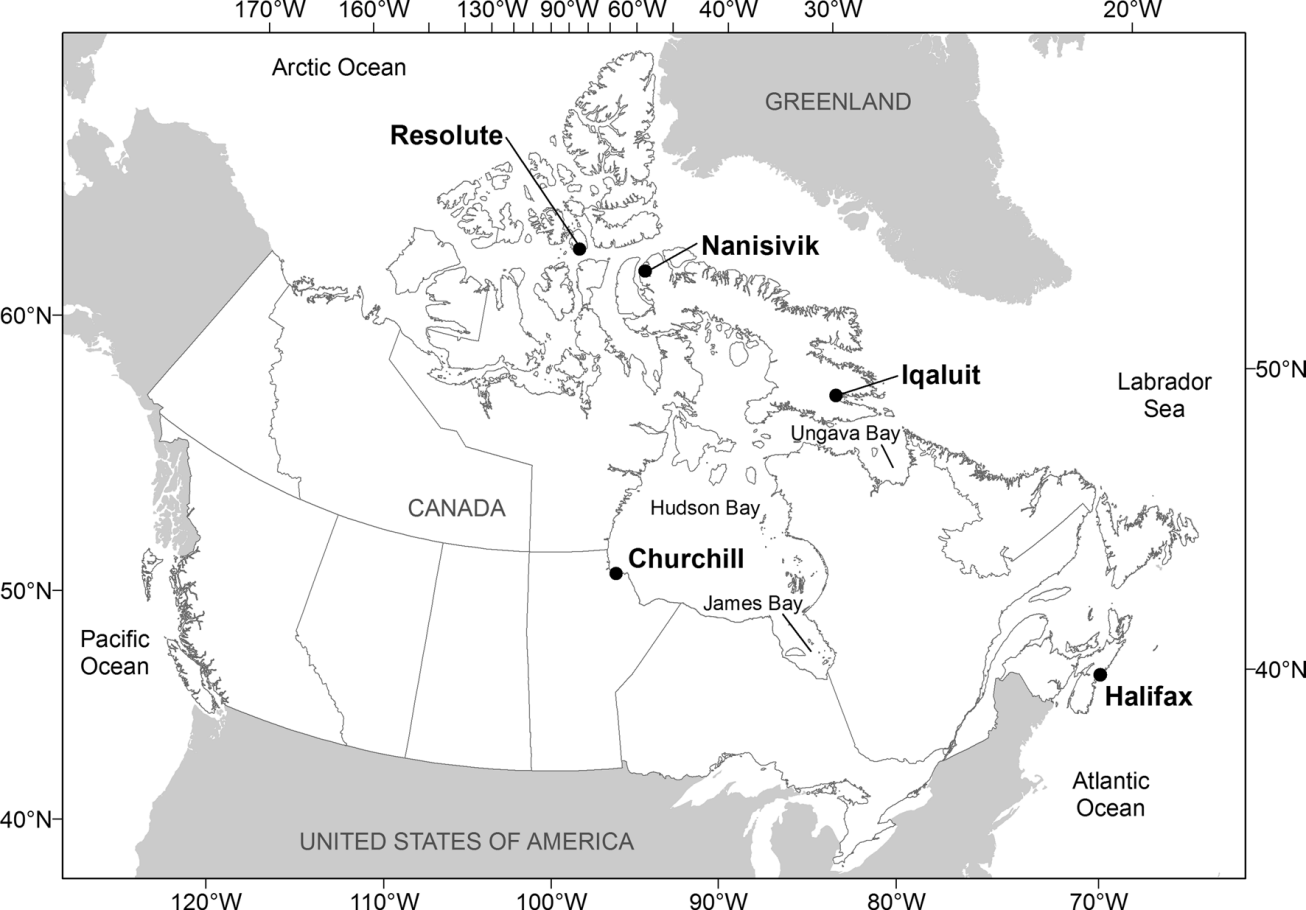
Location of sampling sites, including Halifax (44°39′23′′N, 63°34′44′′W), Iqaluit (63°45′00′′N, 68°31′60′′W), Nanisivik (73°04′00′′N, 84°32′60′′W), Resolute (74°40′60′′N, 94°52′00′′W), and Churchill (58°46′59′′N, 94°13′0′′W)

The Canadian Forces Base Halifax is Canada’s naval base and home port on the Atlantic coast. The dockyard is located on the western side of Halifax Harbour (Fig. 1). The harbour is generally ice-free year-round, though parts of the harbour can develop ice cover in January and February during harsh winters (Canadian Ice Service 2015). Annual mean surface water temperature is 8.5 °C (mean range 1.0–19.6 °C), with annual salinity averaging 30.8 ppt (Keller et al. 2011; Dabbous and Scott 2012).

Sampled ships departed Halifax for one of the following Arctic ports in Canada: Iqaluit, Nanisivik, or Resolute in Nunavut, or Churchill in Manitoba (Fig. 1). Canada’s Arctic covers all Canadian waters north of 60° plus Ungava Bay, Hudson Bay, and James Bay (Canadian Coast Guard 2014; Fig. 1). Iqaluit is situated on the southern coast of Baffin Island at the head of Frobisher Bay. The port is characterized by extensive tidal flats, with a mean tidal range of 7.3–11.6 m during large tides (McCann and Dale 1986). Sea ice typically covers Frobisher Bay, except for an open-water season between July and October (McCann and Dale 1986). Annul mean surface water temperature at Iqaluit is 0.0 °C (mean range 0.0–8.8 °C), with annual salinity averaging 30.7 ppt (Keller et al. 2011). Nanisivik is located at the northern end of Baffin Island on the southern shore of Strathcona Sound off Admiralty Inlet. Strathcona Sound is typically dominated by the presence of land-fast first-year ice about 1.6 m thick, with freeze-up commencing in September and break-up occurring in June (Frederking and Nakawo 1984). Annual mean surface water temperature and salinity at Nanisivik are 0.0 °C (mean range 0.0–7.9 °C) and 7.5 ppt, respectively (Keller et al. 2011). Resolute is situated on the southern coast of Cornwallis Island at the end of Resolute Bay, the waterway into Parry Channel, and the Northwest Passage. Resolute Bay is usually covered by sea ice from October to July (Fortier et al. 2002). Annual mean surface water temperature at Resolute is 0.0 °C (mean range 0.0–6.4 °C), and annual average salinity is 30.1 ppt (Keller et al. 2011). Churchill is located on the western shore of Hudson Bay, a large (0.8 × 10^6^ km^2^) and shallow (mean depth ~ 150 m) inland sea connected to the Arctic Ocean by the Foxe Basin in the north and the Labrador Sea by the Hudson Strait in the east (Saucier et al. 2004). Sea ice covers most of the bay from November to June, with ice-free conditions occurring in the summer (Saucier et al. 2004). Annual mean surface water temperature and salinity at Churchill are 4.3 °C (mean range 2.5–8.1 °C) and 26.3 ppt, respectively (Keller et al. 2011).

### Biofouling survey

We surveyed the hulls of six military ships, including three Kingston-class coastal defence ships and three Halifax-class frigates, immediately before, during (when possible), and after eight round-trip voyages from Halifax to Arctic ports in Canada between July and September of 2008–2012 (Table 1). Each voyage lasted approximately two months. Typical sailing speed of Kingston- and Halifax-class ships is 10–12 and 16 knots, respectively, but all ships generally travelled at 10–12 knots in Arctic waters (M. Fontaine, Department of National Defence, personal communication, 2013). Hulls of all ships were treated with Interspeed 640, a copper-based anti-fouling coating manufactured by International Marine (M. Fontaine, Department of National Defence, personal communication, 2010). Time since last dry-docking (i.e. last application of anti-fouling coating) ranged from two to four years (M. Fontaine, Department of National Defence, personal communication, 2013). Biological sampling was opportunistic, based on availability of ships and dive crews as well as on weather and tidal currents. For each ship, we conducted an underwater survey first at the Canadian Forces Base Halifax, second, when possible at ports in Iqaluit, Nanisivik, Resolute, or Churchill, and finally, upon return to Halifax. Our sample collection and processing methods generally followed those described by Sylvester and MacIsaac (2010), Sylvester and Floerl (2014), and Chan et al. (2015b). Briefly, SCUBA divers inspected and recorded videos or still images of the full length of each ship’s hull during the initial survey in Halifax. Divers collected samples from underwater locations where biofouling was observed using a scraper and re-sealable plastic bags for hard-shelled animals or a sampling syringe with a mounted blade for soft-bodied organisms. We standardized sampling area for each collection using a 20 × 20 cm quadrat. Because each sample inevitably included a volume of ambient port water, divers also collected three water samples of approximately 1 L each at depths corresponding to ships’ waterline, mid-hull, and keel to be used as controls (see below). When possible, we examined samples in sorting trays at the surface to determine the viability of organisms upon collection; however, this analysis was limited to organisms such as amphipods, bivalves, and cirripedes that are large enough to be checked reliably with the naked eye. We sieved biofouling samples through a 45-μm mesh and preserved them in 95% ethanol at room temperature until analysis. We repeated these procedures and collected biofouling samples from the same underwater locations, adjacent to the previously sampled quadrats of individual ships at subsequent surveys in Arctic ports and/or at Halifax. In total, we conducted 20 underwater surveys, including four sets of before–during–after surveys and another four sets of before–after surveys (Table 1). We were not able to conduct surveys in the Canadian Arctic during four voyages owing to logistical constraints. The number of biofouling samples collected per survey ranged from 5 to 18, depending on the extent of biofouling on ships at the initial survey (Table 1). We were able to record videos or still images for only eight surveys; quality of the footage and photographs was inconsistent within sets of before–during–after and before–after surveys. Therefore, we could not confidently estimate percent cover of biofouling for the entire ship. Consequently, we focused our analysis of biofouling assemblages at the quadrat level.

**Table 1:** Summary of information on ship biofouling surveys conducted in this study including voyage ID, ship ID, ship class, sampling time point, and sampling location

Voyage ID	Ship		Sampling time point and location			
	ID	Class	Year	Before Arctic (mid-July)	During Arctic (mid-August)	After Arctic (mid-September)
1	A	KIN	2009	Halifax (10)	(0)	Halifax (10)
2	B	HFX	2009	Halifax (8)	Iqaluit (8)	Halifax (8)
3	A	KIN	2010	Halifax (8)	(0)	Halifax (8)
4	C	KIN	2010	Halifax (10)	Resolute (10)	Halifax (10)
5	D	HFX	2010	Halifax (16)	Nanisivik (16)	Halifax (16)
6	E	KIN	2011	Halifax (5)	(0)	Halifax (5)
7	F	HFX	2011	Halifax (12)	(0)	Halifax (12)
8	F	HFX	2012	Halifax (18)	Churchill (18)	Halifax (18)

Numbers in parentheses indicate the number of sample collected at each survey. No samples were collected, while ships were in the Canadian Arctic for voyages 1, 3, 6, and 7

*HFX* Halifax-class frigate, *KIN* Kingston-class coastal defence ships

We processed all samples under a dissecting microscope in the laboratory following established protocols (Sylvester and MacIsaac 2010; Sylvester et al. 2011; Chan et al. 2015b). We collected a minimum of 30 individuals per morphotype and identified them to the lowest taxonomic level feasible with the assistance of taxonomic experts (see Acknowledgements). We were not able to identify many individuals to the species level; hence, our analysis may underestimate the true species richness of biofouling assemblages and unidentified taxa may obscure differences in species composition among assemblages. We considered species present in control water samples to be members of the port community rather than the biofouling assemblage, thus excluding them from our analysis (Chan et al. 2015b).

We categorized all collected biofouling assemblages into four location groupings: (1) niche areas, (2) bow, (3) stern, and (4) main hull. Niche areas, including sea chest grating, stern tube, rope guard, propeller, and rudder, are topographically complex and protected areas on ships; such locations are particularly vulnerable to biofouling (Coutts and Taylor 2004; Coutts et al. 2010a; Sylvester and MacIsaac 2010). Biofouling organisms at the bow and the stern are subjected to varying degrees of hydrodynamic forces that can influence their survivorship during voyages, with those at the bow experiencing the greatest forces and those at the stern the least when compared to other locations on the hull (Coutts et al. 2010b; Lindholdt et al. 2015). We also classified all biofouling taxa into three categories largely based on motility: (1) mobile invertebrates; (2) sessile and sedentary invertebrates; and (3) algae (modified from Canning-Clode and Sugden 2014). Mobile invertebrates include free-moving invertebrates (e.g. acarines, amphipods, and copepods) that are not affixed to surfaces of the hull. Sessile and sedentary invertebrates are those that attach to hull surfaces with minimal adult movement (e.g. ascidians, bivalves, and cirripedes) and those that inhabit tubes or burrows with occasional movement outside of their dwellings (e.g. spionid polychaetes), respectively. Algae include all algal taxa identified in this study. We did not differentiate those that grow attached to hulls from free-floating ones because most algal taxa were not identified to species level, and thus motility could not be confidently determined.

To determine biogeographic distributions of biofouling taxa, we conducted an extensive literature review of scientific journal publications, taxonomic keys, government reports, and online biodiversity databases and consulted with taxonomic experts (see Table S1 and Acknowledgement). We classified all taxa into three categories: (1) existing: those that have previously been recorded in the Arctic region of Canada; (2) new: those that have not been reported from Canada’s Arctic; and (3) unknown: taxa whose distribution could not be determined because they were not identified to species level. Existing species are presumably native to the port region, but insufficient baseline biodiversity information for Canada’s Arctic coastal systems prevents us from confirming their biogeographic status (Cusson et al. 2007; Goldsmit et al. 2014). New species may include those that are nonindigenous to the Canadian Arctic and those that are native in the port area but have not yet been reported (Goldsmit et al. 2014).

### Statistical analysis

We explored how species richness and abundance of biofouling assemblages on ships varied over time by plotting sampling time point on the *x*-axis and number of all biofouling taxa or total abundance of solitary biofouling taxa per quadrat on the *y*-axis. We examined differences in biofouling assemblage structure before, during, and after Arctic voyages graphically using nonmetric multidimensional scaling (nMDS). We also tested for differences in assemblage structure for matched samples (i.e. those collected at the same hull locations over time) quantitatively using permutational multivariate analysis of variance (PERMANOVA), following recommendations of Anderson et al. (2008) for crossed-nested, unbalanced experimental design with repeated measures. We included time (before, during, and after Arctic), hull location (niche areas, bow, stern, and lateral hulls) nested in ship, and the interaction term between time and hull location as fixed factors and ship as a random variable in our analyses. In the case of significant factors, we conducted permutational pairwise tests on levels of the factors (Anderson et al. 2008). We performed similarity percentage analyses (SIMPER) to identify specific taxa responsible for driving differences in biofouling assemblage structure observed in nMDS and PERMANOVA (Clark and Warwick 2001). To examine whether motility of organisms contributed to temporal variation in biofouling assemblage structure, we conducted similar nMDS and PERMANOVA analyses, separately, for mobile invertebrates, sessile and sedentary invertebrates, and algae.

Initially, we conducted individual analyses for all biofouling taxa and solitary taxa only. For all taxa, we examined similarity among assemblages using presence–absence data measured by the Søresen coefficient so that colonial taxa that are difficult to enumerate could be included. For solitary taxa, we log_10_(*x* + 1)-transformed abundance data and used the Bray–Curtis dissimilarity index as the multivariate distance measure. However, since both approaches provided consistent results, we present results obtained based on presence–absence data only for brevity. All statistical analyses were conducted using PRIMER v6 with the PERMANOVA + add-on (Anderson et al. 2008).

## Results

We identified a total of 293 distinct taxa from biofouling samples, excluding those present in port water (Tables S1, S2, and S3). The majority (73%) of these taxa were mobile invertebrates including acarines, amphipods, chaetognaths, chironomids, cladocerans, copepods, decapods, echinoderms, gastropods, isopods, nematodes, nemerteans, oligochaetes, ostracods, platyhelminths, and free-moving polychaetes, followed by sessile and sedentary taxa (18%) including bivalves, bryozoans, cirripedes, cnidarians, hydrozoans, tube- or burrow-dwelling polychaetes, and tunicates. Algal taxa (9%) were also present in biofouling samples. We classified 54, 58, and 181 taxa as existing, new, and unknown taxa, respectively (Table S1). When considering only samples collected at Canadian Arctic ports, we identified a total of 58 distinct taxa (Table S1). These include six taxa that have not been reported in the Canadian Arctic: the copepod *Harpacticus obscurus*, the cirripede *Amphibalanus improvisus*, and four nematodes *Chromadorina erythrophthalma, Geomonhystera* sp. 1, *Graphonema* sp., and *Prochromadora* sp. 3 (Table 2; Table S1). Individuals of these taxa were also present in samples collected at Halifax prior to Arctic voyages, and in some cases, after Arctic voyages (Table 2). We detected live specimens of *Graphonema* sp. in biofouling samples collected at Iqaluit (Table S1). *A. improvisus* has the potential to survive if propagules are released into Churchill, based on its known temperature and salinity requirements (Fofonoff et al. 2016). Physiological tolerance information was not available for the remaining new taxa; however, based on their occurrences in cold temperate coastal waters, *H. obscurus* may tolerate environmental conditions in Churchill, whereas the potential for *C. erythrophthalma* to survive in Nanisivik is unclear.

**Table 2:** Selected taxa found in biofouling assemblages collected from hulls of six ships before, during, and after round-trip voyages between Halifax and Arctic ports in Canada

Taxon	Sampling time point			Port
	Before Arctic	During Arctic	After Arctic	
Copepoda				
*Harpacticus obscurus*	×	×	×	Churchill
Cirripedia				
* Amphibalanus improvisus*	×	×		Churchill
Nematoda				
* Chromadorina erythrophthalma*	×	×		Nanisivik
* Geomonhystera* sp. 1	×	×	×	Iqaluit, Nanisivik, Resolute
* Graphonema* sp.	×	×		Iqaluit
* Prochromadora* sp. 3	×	×		Iqaluit, Nanisivik

Only new taxa (i.e. those not previously reported from the Canadian Arctic) found in samples collected at Arctic ports are presented. Occurrence of taxa (×) and sampling port in the Arctic are also included

**Fig. 2:**
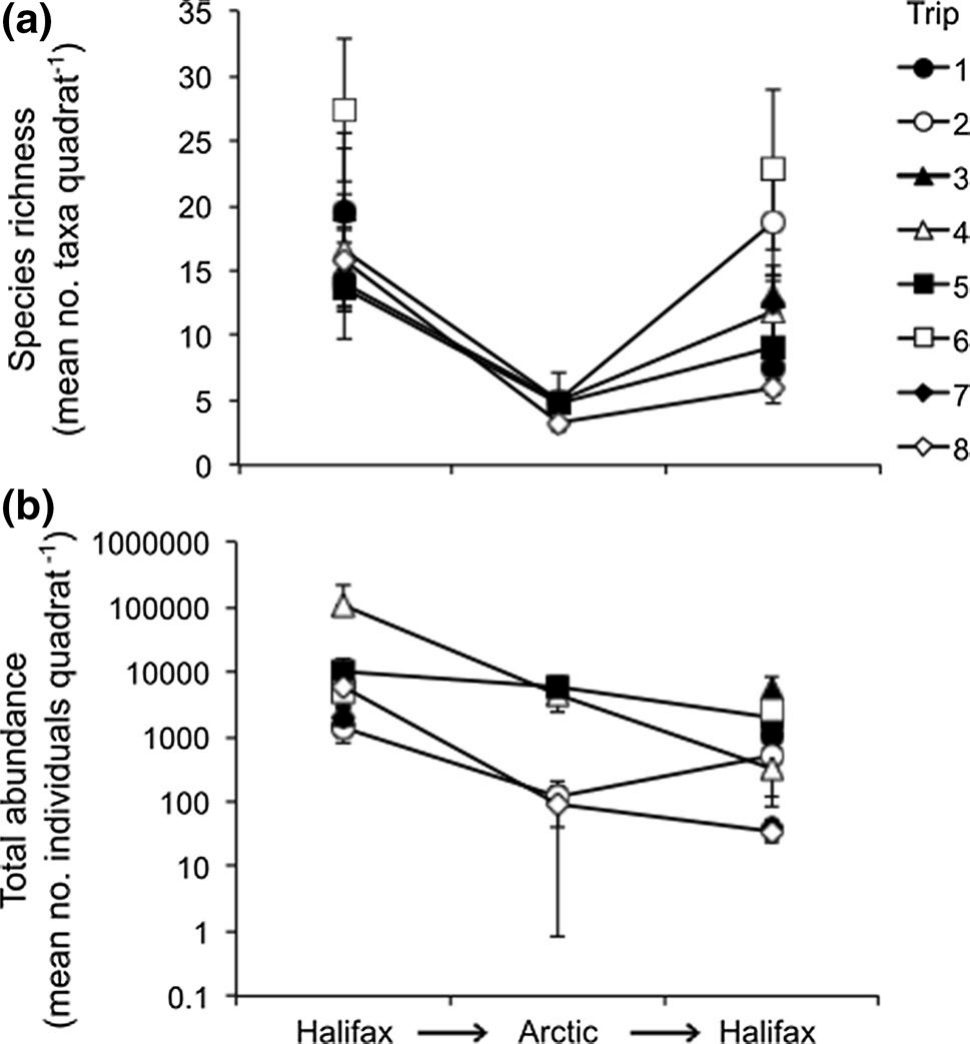
**a** Species richness of all biofouling taxa and **b** total abundance of solitary taxa on military ships before, during, and after round-trip voyages from Halifax to Arctic ports in Canada. Values are presented as mean number of taxa or individuals per quadrat. Standard errors are included. Note the log scale in panel **b**

**Fig. 3:**
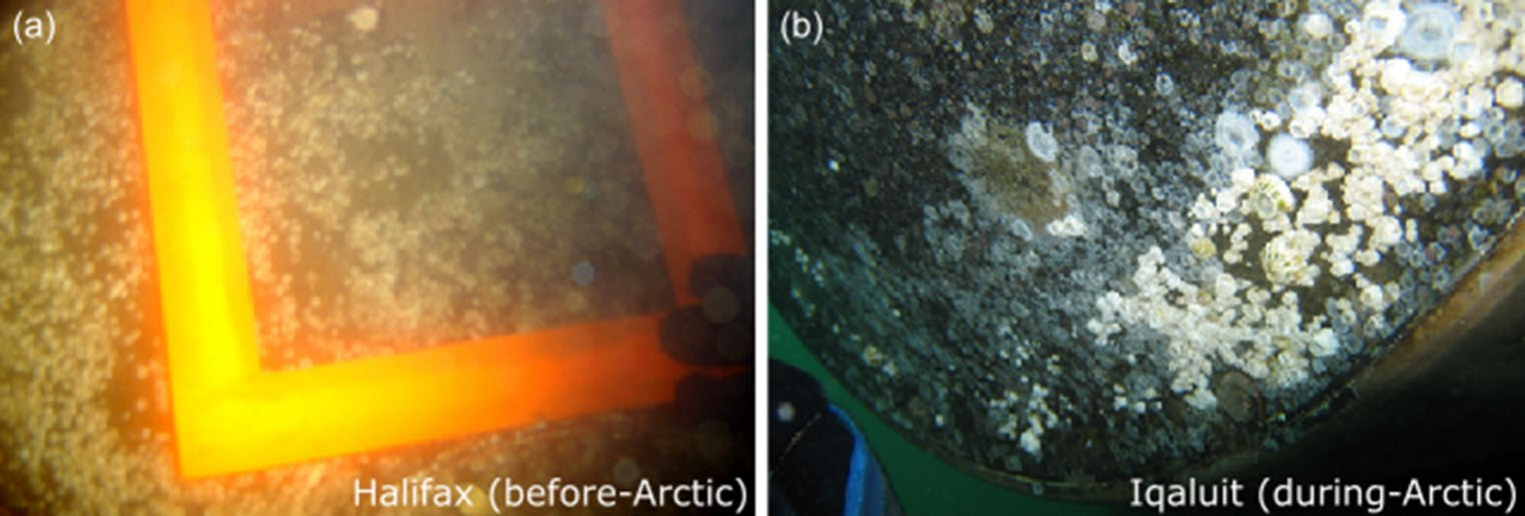
Biofouling assemblages observed at the propeller hub of Ship A in **a** Halifax and **b** Iqaluit, before and during the Arctic transit (voyage 1), respectively

**Fig. 4:**
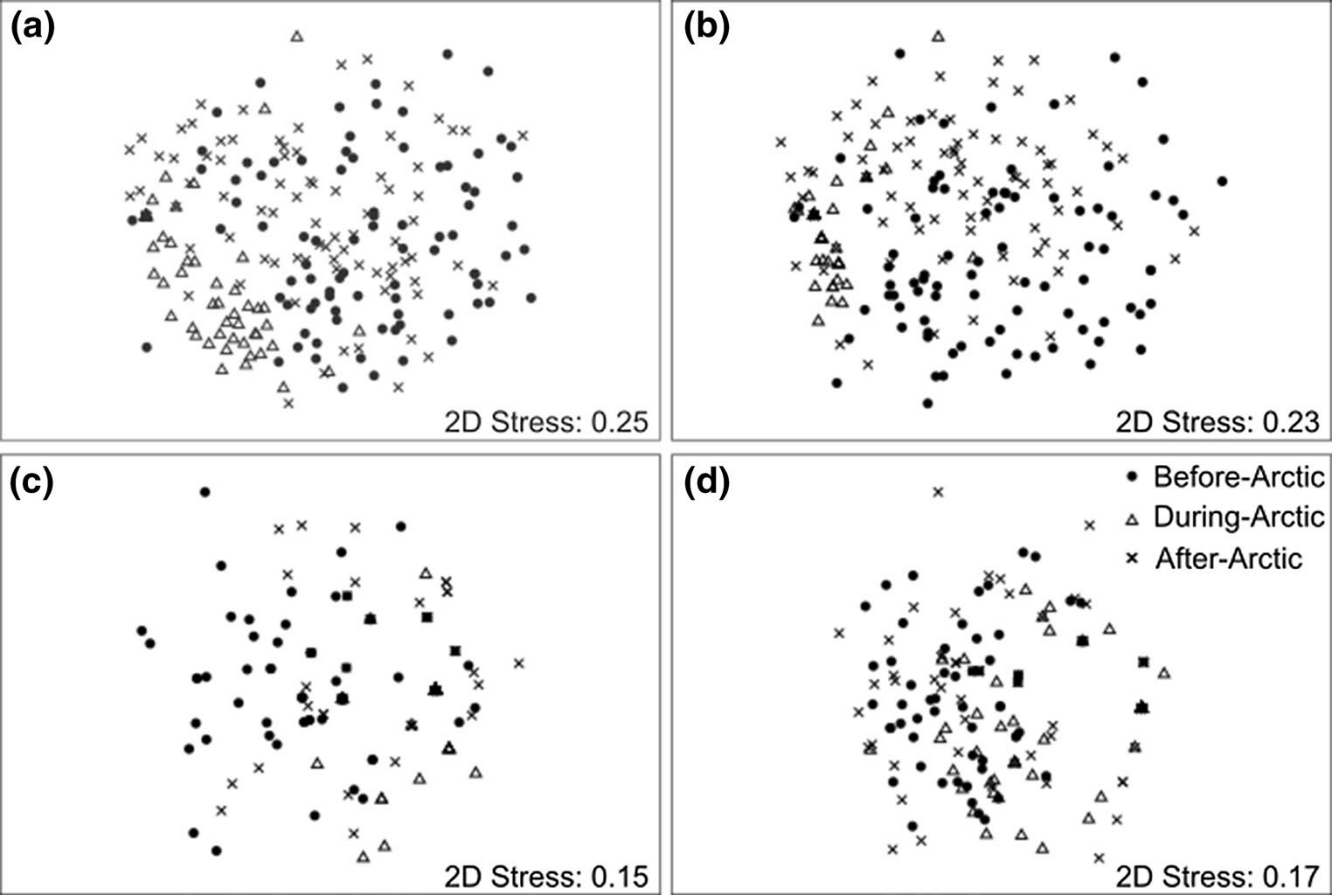
Nonmetric multidimensional scaling (nMDS) plots showing differences in assemblage structure for **a** all taxa, **b** mobile invertebrates, **c** sessile and sedentary invertebrates, and **d** algae on military ships before, during, and after round-trip voyages from temperate to Arctic ports in Canada. Stress, the measure of closeness of fit, is also included. Plots presented were constructed using Søresen index based on presence–absence data of all biofouling taxa

Species richness of biofouling assemblages generally decreased (mean percent loss of 70%) as ships travelled from Halifax to Canadian Arctic ports but recovered (mean percent loss of 27% when compared to original assemblages) after ships returned to Halifax (Fig. 2a; Table S2). In contrast, total abundance of biofouling assemblages, considering solitary taxa only, typically declined over time (mean percent loss of 82 and 55% during and after Arctic voyages, respectively, when compared to original assemblages) (Fig. 2b; Table S2). An example of reduced total abundance of biofouling organisms on the propeller hub of one of the sampled ships upon arrival at Iqaluit is shown in Fig. 3. There was a clear separation in the nMDS plot for biofouling assemblages collected in the Arctic versus those sampled before and after Arctic transits (Fig. 4a). PERMANOVA confirmed that biofouling assemblage structure differed significantly by time and hull location (Table 3a); however, there was no significant interaction between time and hull location indicating that the effect of each variable on assemblage structure was independent of the other (Table 3a). We obtained similar results when conducting separate analyses for mobile invertebrates as well as sessile and sedentary invertebrates (Table 3b; Fig. 4b, c). For algae, samples collected in the Arctic overlapped with those collected in Halifax in the nMDS plot, suggesting that there were no differences in assemblage structure across sampling time points (Fig. 4d). Indeed, only hull location significantly affected structure of algal assemblages (Table 3b).

**Table 3:** Results of permutational multivariate analysis of variance (PERMANOVA) testing the effects of time, hull location, and their interaction (time × hull location) on the structure of biofouling assemblages on ships during round-trip voyages from temperate to Arctic ports in Canada

	d.f.	Presence–absence	
		pseudo-*F*	*P*
(a) *All taxa*			
Time	2	3.43	<0.01
Hull location	19	1.56	<0.01
Time × hull location	28	1.06	0.26
(b) *By motility*			
Mobile invertebrates			
Time	2	4.79	<0.01
Hull location	19	1.42	<0.01
Time × hull location	28	1.07	0.25
Sessile and sedentary invertebrates			
Time	2	3.23	*0.02*
Hull location	19	2.09	<0.01
Time × hull location	28	1.03	0.40
Algae			
Time	2	0.86	0.53
Hull location	19	2.04	<0.01
Time × hull location	28	1.23	0.07

We found significant differences in biofouling assemblage structure between samples collected before and during Arctic transits as well as between those collected during and after voyages (Table 4a). SIMPER analyses revealed that differences in assemblage structure for before versus during Arctic samples were mainly driven by an increase in abundance of the nematode *Chromadorina* sp. 1 (dissimilarity contribution = 9.59%) and decreases in abundance of the copepod *H. obscurus* and the nematode *Geomonhystera* sp. 1 (dissimilarity contributions = 8.97% and 7.22%, respectively). In the case of during versus after Arctic samples, differences in assemblage structure were primarily a result of decreases in abundance of the nematodes *Chromadorina* sp. 1 and *Geomonhystera* sp. 1 (dissimilarity contributions = 13.28 and 8.79%, respectively) and an increase in abundance of the copepod *Tisbe* spp. (dissimilarity contribution = 8.68%). Assemblage structure for samples collected before and after Arctic transits was not significantly different from each other (Table 4a). Separate pairwise comparisons conducted for biofouling assemblages by motility generated similar results (Table 4b).

**Table 4:** Permutational pairwise comparisons of biofouling assemblage similarity between sampling time points (before, during, and after Arctic transits) during round-trip voyages from temperate to Arctic ports in Canada

	Presence–absence
	*P*
(a) *All taxa*	
Before–after	0.22
Before–during	<0.01
During–after	<0.01
(b) *By motility*	
Mobile invertebrates	
Before–after	0.15
Before–during	<0.01
During–after	<0.01
Sessile and sedentary invertebrates	
Before–after	0.10
Before–during	<0.01
During–after	<0.01

Descriptions of data and symbols used are given in Table 2

Pairwise comparisons of similarity in assemblage structure between hull locations revealed no apparent pattern (Table 5a). There were significant differences in assemblage structure between pairs of hull locations for some (e.g. trips 1, 2, 4, and 7) but not all trips (Table 5a). In cases where there were significant differences in assemblage structure between pairs of hull locations, the hull location pairs differed by trips (Table 5a). Similarly, there was no clear pattern when conducting separate pairwise comparisons for individual motility groups (Table 5b).

**Table 5:** Permutational pairwise comparisons of biofouling assemblage similarity between hull locations of ships during round-trip voyages from temperate to Arctic ports in Canada

Presence–absence								
Ship	1	2	1	3	4	5	6	6
Trip	1	2	3	4	5	6	7	8
	*P*							
(a) *All taxa*								
Bow–hull	0.71	–	0.79	0.26	–	–	0.19	0.24
Bow–niche	0.53	–	0.54	–	–	0.56	0.32	0.07
Bow–stern	<0.01	–	–	–	–	–	–	0.17
Hull–niche	–	0.07	0.47	0.02	0.40	0.34	0.04	0.15
Hull–stern	0.15	0.07	–	0.02	–	–	–	0.41
Niche–stern	0.05	<0.01	–	–	–	0.42	–	0.86
(b) *By motility*								
Mobile invertebrates								
Bow–hull	0.57	–	0.78	0.26	–	–	0.30	0.19
Bow–niche	0.73	–	0.64	–	–	0.87	0.30	0.68
Bow–stern	<0.01	–	–	–	–	–	–	0.33
Hull–niche	–	0.09	0.65	0.04	0.62	0.60	0.01	0.77
Hull–stern	0.14	0.51	–	0.11	–	–	–	0.29
Niche–stern	0.10	0.15	–	–	–	0.42	–	0.91
Sessile invertebrates								
Bow–hull	0.69	–	0.35	0.28	–	–	0.37	0.61
Bow–niche	0.69	–	0.49	–	–	0.26	0.33	0.56
Bow–stern	<0.01	–	–	–	–	–	–	0.59
Hull–niche	–	0.14	0.33	0.02	0.14	0.62	0.22	<0.01
Hull–stern	0.19	0.19	–	<0.01	–	–	–	0.26
Niche–stern	0.18	0.01	–	–	–	0.62	–	0.34
Algae								
Bow–hull	0.67	–	0.79	0.49	–	–	0.20	0.15
Bow–niche	0.41	–	0.45	–	–	0.20	0.24	<0.01
Bow–stern	<0.01	–	–	–	–	–	–	0.07
Hull–niche	–	0.39	0.37	0.16	0.25	0.16	0.35	0.08
Hull–stern	0.20	0.01	–	0.01	–	–	–	0.22
Niche–stern	0.02	0.02	–	–	–	0.29	–	0.64

Descriptions of data and symbols used are given in Table 2

## Discussion

This is the first study to characterize temporal changes in biofouling assemblages on ships during transits in the marine Arctic, a system that is currently under elevated invasion threat owing to climate change, resource development, and expanded Arctic shipping (Miller and Ruiz 2014). In general, species richness of biofouling assemblages first decreased and then recovered as ships travelled to and from the Arctic, respectively (Fig. 2a). Conversely, total abundance of biofouling organisms typically declined over time (Fig. 2b). We observed significant differences in assemblage structure between biofouling collected before and during Arctic transits as well as those sampled during and after voyages (Fig. 4). These differences were mainly driven by changes in abundance of copepods (e.g. *Harpacticus obscurus* and *Tisbe* spp.) and nematodes (e.g. *Chromadorina* sp. and *Geomonhystera* sp.). We attribute increases in species richness after Arctic voyages to recolonization by port communities because there were delays of two to three days in resampling ships after their return from the Arctic and because assemblage structure is comparable between before and after Arctic samples. Collectively, our results suggest that biofouling assemblages on ships generally have poor survivorship, about 70 and 82% loss in species richness and total abundance, respectively, during passage in Arctic waters. Previous studies examining the pre- and post-voyage survivorship of biofouling organisms in temperate and Antarctic waters also observed significant losses in percent cover or abundance after transits (Brock et al. 1999; Davidson et al. 2008; Coutts et al. 2010b; Lee and Chown 2009), but not necessarily in species richness (Coutts et al. 2010b; Davidson et al. 2008).

The potential to transport new species, including NIS, to the Arctic via ship biofouling is considerable, as six taxa that have not been reported from the Canadian Arctic including the copepod *Harpacticus obscurus*, the cirripede *Amphibalanus improvisus*, and four nematodes, *Chromadorina erythrophthalma*, *Geomonhystera* sp., *Graphonema* sp., and *Prochromadora* sp. appeared to survive transits from Halifax to Arctic ports, and in some cases endured return trips from the Arctic as well. We were able to confirm the presence of live individuals for *Geomonhystera* sp. at Iqaluit. *H. obscurus* and *A. improvisus* could potentially survive if introduced into the recipient Arctic port, Churchill, based on known distributions and thermal and salinity tolerances, respectively (OBIS 2015; Fofonoff et al. 2016). *A. improvisus* is of particular concern because this species is a pervasive, high-impact NIS (Molnar et al. 2008). If successfully established, *A. improvisus* may compete with native species for food and space and alter habitat and trophic structure by filtering phytoplankton, remineralizing nutrients, increasing the clarity of water, and promoting the growth of macrophytes in invaded habitats (Fofonoff et al. 2016). Live specimens of *A. improvisus* were previously found on international commercial ships arriving at Churchill (Chan et al. 2015b). Our finding adds to growing evidence documenting that temperate biofouling taxa, including NIS, are capable of surviving transits in polar waters (Lewis et al. 2004, 2006; Lee and Chown 2007, 2009; Chan et al. 2015b; Hughes and Ashton 2016). For example, Lewis et al. (2004) found 72% of original biofouling assemblage, including invasive taxa, survived a voyage from Western Australia to Heard Island (sub-Antarctic) and then back to Australia (Tasmania). More recently, Hughes and Ashton (2016) reported viable individuals of gooseneck (*Conchoderma auritum*) and balanomorph cirripedes on a research ship that travelled from the UK to the Antarctic Peninsula. In contrast, the likelihood of transferring NIS on ship hulls from Arctic to temperate ports seems low. Although there were biofouling taxa (24 species in total) present in after Arctic samples that were not recorded before Arctic, none are nonindigenous to Halifax.

While biofouling assemblage structure varied by hull location, temporal variation in assemblage structure during Arctic voyages was independent of location on the hull. A number of studies have found significant differences in biofouling species richness and percent cover across underwater locations on ship hulls, with niche areas such as sea chest gratings, propellers, and rudders tending to be more heavily fouled than the main hull itself (e.g. Coutts and Taylor 2004; Davidson et al. 2009; Sylvester and MacIsaac 2010; Chan et al. 2015b). Such variation in biofouling patterns on ships has been attributed to varying effectiveness of anti-fouling paint, exposure to hydrodynamic flow, availability of sunlight across hull locations, or a combination of these factors (Coutts and Taylor 2004). Anti-fouling coatings are usually not applied effectively or at all to niche areas because these locations are difficult to access and efficacy is often compromised owing to insufficient water flow (e.g. rope guards) or extreme turbulence (e.g. bulbous bows) (Coutts and Taylor 2004). Reduced hydrodynamic flow in niche areas allows a wide variety of biofouling taxa to settle and remain attached during transit, with the exception of bulbous bows and propellers, where only hydrodynamic-insensitive taxa (e.g. brown and green algae and cirripedes) survive owing to strong dislodgement forces (Coutts and Taylor 2004). In addition, some niche areas, including bulbous bows, rope guards, and rudders, favour colonization of algal taxa because of exposure to sunlight (Coutts and Taylor 2004). We were not able to identify biofouling hot spots, however, because fouling pattern varied widely across ships, likely a result of varying voyage histories prior to our surveys. Voyage history is known to influence the nature and extent of biofouling on ships (Coutts 1999; Sylvester et al. 2011; McCollin and Brown 2014). Interestingly, hull location did not contribute to temporal changes in assemblage structure. Notably, niche areas did not provide protection for biofouling taxa from transport conditions during Arctic voyages. This stands in contrast to previous studies that reported biofouling taxa surviving transit in Antarctic waters in sea chests and near intake pipes of ships but not on other hull locations (Lee and Chown 2007; Hughes and Ashton 2016).

Surprisingly, responses in assemblage structure for mobile versus sessile and sedentary invertebrates were similar. Sessile and sedentary taxa are thought to be more successful in remaining affixed to hull surfaces during voyages than mobile taxa because they possess biomechanical properties that can enhance attachment strength and/or reduce drag (Coutts et al. 2010b; Clarke Murray et al. 2012). Examples of these biomechanical properties include byssal threads of bivalves, adhesive substances secreted at the base of ascidians and cirripedes, hard calcareous shells of bivalves and cirripedes, protective tubes of sabellid and serpulid polychaetes, low-profile and encrusting forms of colonial ascidians and bryozoans, and flexible stalks of solitary ascidians (Coutts et al. 2010a, b; Clarke Murray et al. 2012). It is likely that gregarious settlement of sessile and sedentary invertebrates and macroalgae in complex biofouling communities provides structural habitat and protection for mobile taxa against hydrodynamic forces, thereby minimizing their susceptibility to dislodgement during transport and obscuring differences in en route survivorship between the two motility groups (Lewis et al. 2006; Davidson et al. 2009).

Algae appear to be more tolerant of transport conditions typical of Arctic voyages than are mobile, sessile, and sedentary invertebrates. Biofouling algae also possess biomechanical features that allow them to colonize and remain attached to hull surfaces of moving ships. For instance, microalgae such as the diatom *Amphora* spp., glue to substrata by producing extracellular polymeric substances that form adhesive cell coatings, pads, stalks, and films (Callow and Callow 2002; Molino and Wetherbee 2008). Attached algal cells then divide and proliferate, forming dense colonies (i.e. biofilms and slimes) that have very high attachment strength (Callow and Callow 2002; Molino and Wetherbee 2008). In addition, the morphology of algal colonies allows cell masses to lie within the boundary layer, a layer between the surface and water with no net movement, thereby avoiding exposure to hydrodynamic flow and dislodgement from the hull (Molino and Wetherbee 2008). Furthermore, algal cells are capable of gliding on attached substratum by depositing mucilaginous materials, allowing them to migrate to more suitable areas when biotic and abiotic conditions deteriorate at initial attachment sites (Molino and Wetherbee 2008). Macroalgae, including *Ulva* sp., attach to substrata by means of a glycoprotein adhesive secreted by settling spores, which forms a strongly adhesive gel-like pad (Evans 1981; Callow and Callow 2002). In addition, common biofouling macroalgae are capable of tolerating wide fluctuations in environmental conditions that often occur during ship voyages (Evans 1981; Carlton and Hodder 1995; Lewis et al. 2004). They are also resilient to mechanical stress owing to their ability to regenerate from the basal part of the thallus and from detached fragments following breakage (Evans 1981). However, this finding should be interpreted with caution, as the low taxonomic resolution for algal taxa may obscure the observed pattern.

Responses in assemblage structure may be attributed to fluctuations in water temperature and salinity, hydrodynamic forces, and ice scouring. Temperature and salinity are fundamental factors affecting survival, growth, and reproduction of aquatic invertebrates and algae (Hauton 2016; Whiteley and Mackenzie 2016). Drastic changes in either of these variables can cause mortality of organisms (Hauton 2016; Whiteley and Mackenzie 2016). Typical summer water temperature at Halifax is about 13 °C, much warmer than the Canadian Arctic ports which vary from around 0 °C for Nanisivik, 2 °C for Resolute, 5 °C for Iqaluit to 10 °C for Churchill (Locarnini et al. 2013). Therefore, transits between Halifax and Arctic ports could expose biofouling assemblages to temperature variation as great as 13 °C. Differences in annual salinity between Halifax and Arctic ports are less extreme: about 30 ppt at Halifax, Iqaluit, and Resolute and around 24 ppt at Nanisivik and Churchill (Keller et al. 2011). Therefore, temperature may play a more important role than salinity in survivorship of biofouling assemblages during Arctic voyages. Hydrodynamic forces can also contribute to survival of biofouling assemblages during transit (Coutts et al. 2010a; Clarke Murray et al. 2012). Drag, lift, and accelerative forces acting on biofouling assemblages can dislodge organisms from ships during voyages (Coutts et al. 2010a; Clarke Murray et al. 2012). Previous studies have found that extent of biofouling is inversely related to sailing speed because hydrodynamic force is greatest at high sailing speeds (~20 knots) (Coutts et al. 2010a, b; Sylvester et al. 2011). Although ships sampled during this study sailed at relatively slow speeds (10–12 knots), the hydrodynamic forces may have been sufficient to cause detrimental effects on biofouling taxa. Coutts et al. (2010a, b) reported decline in species richness and percent cover of biofouling on ships travelling at 8–10 knots, although significant reductions occurred primarily on faster sailing ships (14–21.5 knots). Furthermore, ice scouring (i.e. mechanical abrasion of sea ice on hulls) can negatively impact and remove biofouling assemblages. A number of studies found that biofouling coverage diminished dramatically after ships transited through sea ice (Lewis et al. 2004; Lee and Chown 2009; Hughes and Ashton 2016); however, our sampled ships avoided contact with sea ice during Arctic voyages (M. Fontaine, Department of National Defence, personal communication, 2013). Therefore, effects of ice scouring on biofouling assemblages were not observed in this study. Unfortunately, we could not confirm and quantify the effects of these selective pressures on the survivorship of biofouling assemblages during Arctic voyages because in situ measurements of environmental and sailing conditions were not available.

The presence of planktonic species (21 species in total) in biofouling samples after the exclusion of taxa present in control port water samples is interesting. It is unclear whether these species were members of the plankton community in ports or the biofouling assemblage on ships. It is possible that they are local planktonic species and that our control water samples (see Methods) were not sufficient to account for them in our analyses. If this is the case, their presence in biofouling samples may inflate species richness and abundance estimations. To further investigate this point, we compared species identified in this study to zooplankton species detected in water samples collected at Canadian Arctic ports using metabarcoding with >97% sequence similarity threshold in Basic Local Alignment Search Tool (BLAST) searches (Chain et al. 2016). The list presented by Chain et al. (2016) is the most comprehensive for zooplankton in the Canadian Arctic that we are aware of. We found only five of the 21 species recorded in Chain et al. (2016). Three of the 16 remaining species have not previously been reported from the Canadian Arctic. Given the uncertainty associated with the origin of these planktonic species, we included them in our analyses to err on the conservative side. Sylvester and MacIsaac (2010) and Sylvester et al. (2011) also observed planktonic species in ship biofouling samples. We argue that the prevalence of planktonic species in biofouling assemblages on ships might have been overlooked in past studies focused on macroinvertebrates or organisms >1 mm (e.g. Davidson et al. 2008; Coutts et al. 2010a, b; Hughes and Ashton 2016). Further studies are required to examine the importance of planktonic organisms in biofouling assemblages.

## Conclusions

Ship biofouling is a major transport vector of NIS globally and may become increasingly important in the Arctic owing to climate warming, resource development, and expansion of Arctic shipping. In this study, we characterized temporal changes in biofouling assemblages on military ships during round-trip voyages from temperate to Arctic ports in Canada. While our results suggested that en route survivorship of biofouling organisms during Arctic voyages is generally poor, the risk of transporting NIS to the Arctic via ship biofouling still exists. Several taxa new to the Canadian Arctic appear to have survived passage in Arctic waters; two of these taxa have the potential to survive if propagules are released into the port environment. We recognize that introduction risk associated with ship biofouling could be refined by quantifying the abundance and richness of NIS actually released into port waters; however, such analyses are very challenging logistically and not feasible in this study. Nonetheless, we demonstrated that ship biofouling is an active vector transporting viable NIS to the Arctic. Improvement in vector management strategies, such as increasing the frequency of hull cleaning and development of new anti-fouling technology, could serve to minimize ship biofouling risk in the Arctic.

## Electronic supplementary material

Below is the link to the electronic supplementary material.

Supplementary material 1 (XLSX 317 kb)

Supplementary material 2 (DOC 501 kb)
