# Supplementary material for: Survival of ship biofouling assemblages during and after voyages to the Canadian Arctic
Source: Mar Biol. 2016 Nov 11;163(12):250. doi: 10.1007/s00227-016-3029-1 (PMC5106487; doi:10.1007/s00227-016-3029-1)
Supplement: Supplementary file 2 — Supplementary material 2 (DOC 501 kb) [file 227_2016_3029_MOESM2_ESM.doc]

Survival of ship biofouling assemblages during and after voyages to the Canadian Arctic

Farrah T. Chan^1,2*^, Hugh J. MacIsaac^1^, and Sarah A. Bailey^2^

^1^Great Lakes Institute for Environmental Research, University of Windsor,

Windsor, Ontario, N9B 3P4, Canada

^2^Great Lakes Laboratory for Fisheries and Aquatic Sciences, Fisheries and Oceans Canada, Burlington, Ontario, L7S 1A1, Canada

*Corresponding author: email: farrah.chan@dfo-mpo.gc.ca;

phone: (+1) 905-336-4862; fax: (+1) 905-336-6437

**Table S1** List of taxa found in biofouling assemblages collected from hulls of six military ships during eight round-trip voyages from temperate (Halifax, Nova Scotia) to Arctic (Iqaluit, Nanisivik, or Resolute in Nunavut or Churchill in Manitoba) ports in Canada. Occurrence of taxa at Arctic ports (x), presence of live specimens in the Arctic (y = yes), distribution category (E = existing, N = new, U = unknown; see text for descriptions of categories), and references used are also included. * indicates planktonic species that were not found in port water samples examined in this study but were detected in those collected by Chain et al. (2016) using metabarcoding with > 97% sequence similarity threshold in Basic Local Alignment Search Tool (BLAST) searches. † denotes planktonic species that were absent from water samples examined in this study and in Chain et al. (2016).

| **Taxon** | **Iqaluit** | **Nanisivik** | **Resolute** | **Churchill** | **Category** | **References** |
| --- | --- | --- | --- | --- | --- | --- |
| **Acari** |  |  |  |  |  |  |
| Acari |  |  |  |  | U |  |
| *Anomalohalacarus* sp. |  |  |  |  | U |  |
| *Arhodeoporus* sp. |  |  |  |  | U |  |
| Halacaridae |  |  |  |  | U |  |
| *Halacarus* sp. |  |  |  |  | U |  |
| *Thalassarachna* sp. |  |  |  |  | U |  |
| **Algae** |  |  |  |  |  |  |
| *Amphora* spp. |  |  |  | x | U |  |
| Bacillariophyceae (chained; *Fragilaria/Synedra* sp.) | x | x | x | x | U |  |
| Bacillariophyceae (free-living) | x |  |  |  | U |  |
| Bacillariophyceae (mat) |  |  |  | x | U |  |
| Bacillariophyceae (tube-dwelling) | x |  |  | x | U |  |
| *Ceratium arcticum*† |  |  |  |  | E | Okolodkov1999; AlgaeBase 2016; OBIS 2016 |
| *Ceratium fusus*† |  |  |  |  | N | Okolodkov1999; AlgaeBase 2016; OBIS 2016; WoRMS 2016 |
| *Ceratium horridum*† |  |  |  |  | N | AlgaeBase 2016; OBIS 2016; WoRMS 2016 |
| *Ceratium* sp. | x | x |  |  | U |  |
| Chlorella | x | x | x |  | U |  |
| *Cladophora* sp. |  |  |  |  | U |  |
| Cyanobacteria | x |  |  |  | U |  |
| Dictyochophyceae |  |  |  | x | U |  |
| Dinoflagellata |  | x |  |  | U |  |
| *Dinophysis* sp. |  |  |  |  | U |  |
| *Diploneis* sp. | x |  |  |  | U |  |
| *Ectocarpus* sp. | x | x | x | x | U |  |
| *Grammatophora* sp. |  |  |  |  | U |  |
| *Licmophora* sp. |  | x | x |  | U |  |
| *Polysiphonia* sp. |  |  |  |  | U |  |
| *Prorocentrum micans*† |  |  |  | x | E | Roff and Legendre 1986; OBIS 2016 |
| *Prorocentrum* sp. |  |  |  |  | U |  |
| *Protoperidinium* sp. |  |  |  |  | U |  |
| *Pylaiella* sp. |  |  |  | x | U |  |
| *Ulothrix* sp. | x | x | x | x | U |  |
| *Ulva* sp. 1 |  | x | x | x | U |  |
| *Ulva* sp. 2 | x | x | x | x | U |  |
| **Amphipoda** |  |  |  |  |  |  |
| *Ampithoe valida* |  |  |  |  | N | Pilgrim and Darling 2010; EOL 2016; Fofonoff et al. 2016; WoRMS 2016; WRIMS 2016 |
| Ampithoidae |  |  |  |  | U |  |
| *Apocorophium acutum* |  |  |  |  | N | EOL 2016; Fofonoff et al. 2016; OBIS 2016; WoRMS 2016; WRIMS 2016 |
| *Apocorophium* sp. |  |  |  |  | U |  |
| *Apohyale* sp. |  |  |  |  | U |  |
| *Calliopius laeviusculus* |  |  |  |  | E | Shoemaker 1920; Steele 1961; Bousfield 1973; Shih et al. 1971; Atkinson and Wacasey 1989a,b; Stewart and Lockhart 2005 |
| *Caprella equilibra* |  |  |  |  | N | Foster et al. 2004; EOL 2016; Marine Species Identification Portal 2016; SLB 2016; WoRMS 2016 |
| *Caprella mutica* |  |  |  |  | N | Molnar et al. 2008; EOL 2016; Fofonoff et al. 2016; WoRMS 2016; Marine Species Identification Portal 2016 |
| *Caprella* sp. |  |  |  |  | U |  |
| Caprellidae |  |  |  |  | U |  |
| Corophiidae |  |  |  |  | U |  |
| *Crassicorne sp.* |  |  |  |  | U |  |
| *Crassicorophium bonellii* |  |  |  |  | N | Bousfield 1973; OBIS 2016; Marine Species Identification Portal 2016; WoRMS 2016; WRIMS 2016 |
| *Crassicorophium crassicorne* |  |  |  |  | E | Goldsmit et al. 2014; Marine Species Identification Portal 2016; WoRMS 2016 |
| *Dexamine thea* |  |  |  |  | N | EOL 2016; OBIS 2016; Marine Species Identification Portal 2016; WoRMS 2016 |
| *Gammarellus angulosus* |  |  |  |  | N | Molnar et al. 2008; EOL 2016; Marine Species Identification Portal 2016; WoRMS 2016; WRIMS 2015 |
| Gammaridea |  |  |  |  | U |  |
| *Gammarus oceanicus* |  |  |  |  | E | Atkinson and Wacasey 1989a,b; Stewart and Lockhart 2005; Goldsmit et al. 2014 |
| *Gammarus* sp. | x (y) |  |  |  | U |  |
| *Gnathopleustes* sp. |  |  |  |  | U |  |
| Ischyroceridae |  |  |  |  | U |  |
| *Ischyrocerus* sp. |  |  |  |  | U |  |
| *Jassa marmorata* |  |  |  |  | N | Bousfield 1973; Molnar et al. 2008; Pilgrim and Darling 2010; ARMS 2016; EOL 2016; Fofonoff et al. 2016; WRIMS 2016 |
| *Jassa* sp. |  |  |  | x | U |  |
| *Monocorophium acherusicum* |  |  |  |  | N | Molnar et al. 2008; EOL 2016; Fofonoff et al. 2016; WoRMS 2016; WRIMS 2016 |
| *Monocorophium insidiosum* |  |  |  |  | N | Molnar et al. 2008; EOL 2016; Fofonoff et al. 2016; WoRMS 2016; WRIMS 2016 |
| *Monocorophium* sp. |  |  |  |  | U |  |
| Pleustidae sp. |  |  |  |  | U |  |
| *Pontogeneia inermis* |  |  |  |  | E | Shoemaker 1920; Shih et al. 1971; Atkinson and Wacasey 1989b; Stewart and Lockhart 2005 |
| *Stenothoidae sp.* |  |  |  |  | U |  |
| **Bivalvia** |  |  |  |  |  |  |
| Bivalvia (juveniles) | x (y) | x | x |  | U |  |
| Cardiidae |  |  |  |  | U |  |
| *Hiatella arctica* |  | x |  |  | E | Atkinson and Wacasey 1989b; Stewart and Lockhart 2005; EOL 2016; Marine Species Identification Portal 2016 |
| Modiolus |  |  |  |  | U |  |
| Myidae |  |  |  |  | U |  |
| Mytilidae | x | x |  | x | U |  |
| *Mytilus edulis* | x (y) |  |  | x | E | Atkinson and Wacasey 1989a,b; Stewart and Lockhart 2005; OBIS 2016 |
| *Mytilus* sp. |  |  |  | x | U |  |
| *Pododesmus* sp. |  |  |  |  | U |  |
| Solenoidea |  |  |  |  | U |  |
| Thyasiridae |  |  |  |  | U |  |
| **Bryozoa** |  |  |  |  |  |  |
| *Alcyonidium polyoum* |  |  |  |  | N | Neves and da Rocha 2008; EOL 2016; OBIS 2016; WoRMS 2016 |
| *Callopora craticula* |  |  |  |  | E | Stewart and Lockhart 2005; OBIS 2016 |
| Campanulariidae |  |  |  |  | U |  |
| *Celleporella hyalina* |  |  |  |  | E | Goldsmit et al. 2014; OBIS 2016 |
| *Electra pilosa* |  |  |  |  | N | Yorke and Metaxas 2011; DAISIE 2016; EOL 2016; OBIS 2016; WoRMS 2016 |
| **Chaetognatha** |  |  |  |  |  |  |
| Chaetognatha |  |  |  |  | U |  |
| **Chironomidae** |  |  |  |  |  |  |
| Chironomidae |  |  |  |  | U |  |
| *Cricotopus* sp. |  |  |  |  | U |  |
| *Telmatogeton* sp. |  |  |  |  | U |  |
| **Cirripedia** |  |  |  |  |  |  |
| *Amphibalanus amphitrite* |  |  |  |  | N | Newman and Ross 1976; Molnar et al. 2008; EOL 2016; Fofonoff et al. 2016; OBIS 2016 |
| *Amphibalanus improvisus* |  |  |  | x | N | Newman and Ross 1976; Molnar et al. 2008; Carlton et al. 2011; Fofonoff et al. 2016; OBIS 2016 |
| *Amphibalanus* spp. |  |  |  |  | U |  |
| Balanidae sp. |  |  |  | x | U |  |
| Balanoidea sp. |  |  |  |  | U |  |
| *Balanus balanus* | x (y) |  |  |  | E | Atkinson and Wacasey 1989a,b; Stewart and Lockhart 2005; OBIS 2016 |
| *Balanus crenatus* | x (y) |  |  |  | E | Atkinson and Wacasey 1989a; Stewart and Lockhart 2005; Goldsmit et al. 2014; OBIS 2016 |
| *Semibalanus balanoides* |  |  |  |  | E | ARMS 2015; EOL 2016; WoRMS 2016 |
| **Cladocera** |  |  |  |  |  |  |
| *Daphnia* sp. |  |  |  |  | U |  |
| *Evadne nordmanni*† |  |  |  |  | E | Shih et al. 1971; ARMS 2015; OBIS 2016 |
| *Pleopis polyphaemoides* |  |  |  |  | N | Durbin et al. 2008; Marine Species Identification Portal 2016; OBIS 2016 |
| Podonidae |  |  |  |  | U |  |
| *Polyphemus pediculus*† |  |  |  |  | E | Jeffery et al. 2011; EOL 2016 |
| **Cnidaria** |  |  |  |  |  |  |
| Actiniaria |  |  |  |  | U |  |
| **Copepoda** |  |  |  |  |  |  |
| *Acartia hudsonica*† |  |  |  |  | E | OBIS 2016; WoRMS 2016 |
| *Acartia longiremis*† |  |  |  |  | E | Willey 1931; Shih et al. 1971; Roff and Legendre 1986; Stewart and Lockhart 2005 |
| *Acartia* spp. |  |  |  |  | U |  |
| *Alteutha oblonga* |  |  |  |  | N | Veit-Köhler and Fuentes 2007; OBIS 2016; WoRMS 2016 |
| *Ameira longipes* |  |  |  |  | E | Shih et al. 1971; Ólafsson et al. 2001; ARMS 2016; |
| *Ameira* sp. |  |  |  |  | U |  |
| Ameiridae |  |  |  |  | U |  |
| *Amonardia normani* |  |  |  |  | N | de Souza Santos and Castel 2013; EOL 2016; WoRMS 2016 |
| *Amphiascoides* sp. |  |  |  |  | U |  |
| Ancorabolidae |  |  |  |  | U |  |
| Calanoida |  |  |  |  | U |  |
| *Calanus finmarchicus** |  |  |  |  | E | Willey 1931; Grainger 1963; Shih et al. 1971; Roff and Legendre 1986; Stewart and Lockhart 2005; OBIS 2016 |
| *Calanus glacialis*† |  |  |  |  | E | Shih et al. 1971; Grainger 1963; Roff and Legendre 1986; Stewart and Lockhart 2005; OBIS 2016 |
| *Calanus* sp. |  | x |  |  | U |  |
| *Centropages hamatus*† |  |  |  |  | E | Stewart and Lockhart 2005; Stewart and Howland 2009; OBIS 2016; WoRMS 2016 |
| *Centropages* sp. |  |  |  |  | U |  |
| *Centropages typicus*† |  |  |  |  | N | Beaugrand et al. 2007; OBIS 2016; WoRMS 2016 |
| Cyclopoida |  |  |  |  | U |  |
| *Dactylopodamphiascopsis latifolius* |  |  |  |  | E | Shih et al. 1971; EOL 2016 |
| *Dactylopusia* spp. |  |  |  |  | U |  |
| *Dactylopusia tisboides* |  |  |  |  | E | Shih et al. 1971; Stewart and Lockhart 2005 |
| *Dactylopusia vulgaris* |  |  |  |  | E | Shih et al. 1971; Stewart and Lockhart 2005 |
| *Ectinosoma* sp. |  |  |  |  | U |  |
| Ectinosomatidae |  |  |  |  | U |  |
| *Eurytemora herdmani*† |  |  |  |  | E | Steele 1961; Grainger 1968; Shih et al. 1971; Roff and Legendre 1986; Stewart and Lockhart 2005; OBIS 2016 |
| *Euterpina acutifrons** |  |  |  |  | E | ARMS 2016; OBIS 2016 |
| Harpacticoida |  |  |  |  | U |  |
| *Harpacticus chelifer* |  |  |  |  | E | Stewart and Lockhart 2005; WoRMS 2016 |
| *Harpacticus septentrionalis* |  |  |  |  | N | Cordell 2001; WoRMS 2016 |
| *Harpacticus* sp. |  |  |  |  | U |  |
| *Harpacticus* sp.-*obscurus* group |  |  |  | x | N | Cordell 2001; OBIS 2016; WoRMS 2016 |
| *Harpacticus* sp.-*uniremis* group |  |  |  |  | E | Steele 1961; Shih et al. 1971; Stewart and Lockhart 2005; WoRMS 2016 |
| *Heterolaophonte discophora* |  |  |  |  | E | Shih et al. 1971; Stewart and Lockhart 2005; WoRMS 2016 |
| *Heterolaophonte mendax* |  |  |  |  | N | EOL 2016; WoRMS 2016 |
| *Heterolaophonte* sp. |  |  |  |  | U |  |
| *Laophonte depressa* |  |  |  |  | E | Shih et al. 1971; EOL 2016 |
| Laophontidae |  |  |  | x | U |  |
| *Mesochra* sp. |  |  |  |  | U |  |
| *Microsetella norvegica*† |  |  | x |  | E | Shih et al. 1971; OBIS 2016; WoRMS 2016 |
| *Monstrilla* sp*.* |  |  |  |  | U |  |
| *Nitokra lacustris* |  |  |  |  | N | Rhodes 2003; WoRMS 2016 |
| *Oithona atlantica*† |  |  | x |  | E | OBIS 2016; WoRMS 2016 |
| *Oithona similis** |  | x |  |  | E | Steele 1961; Shih et al. 1971; Roff and Legendre 1986; Stewart and Lockhart 2005 |
| *Oncaea* spp. |  | x |  |  | U |  |
| *Paracalanus parvus*† |  |  |  |  | E | Shih et al. 1971; Stewart and Lockhart 2005; OBIS 2016 |
| *Paradactylopodia brevicornis* |  |  |  |  | E | Shih et al. 1971; ARMS 2016 |
| *Paralaophonte brevirostris* |  |  |  |  | N | EOL 2016; WoRMS 2016 |
| *Paralaophonte perplexa* group |  |  |  |  | E | Shih et al. 1971; Brunel et al. 1998; Stewart and Lockhart 2005; EOL 2016 |
| *Paraleptastacus* sp. |  |  |  |  | U |  |
| *Paramphiascella hispida* |  |  |  |  | E | Shih et al. 1971; EOL 2016 |
| *Paramphiascopsis* sp. |  |  |  |  | U |  |
| *Parastenhelia spinosa* |  |  |  |  | E | Shih et al. 1971; Johnson and Scheibling 1986; ARMS 2016 |
| *Parathalestris harpactoides* |  |  |  |  | N | EOL 2016; GBIF 2016; WoRMS 2016 |
| *Paronychocamptus huntsmani* |  |  |  |  | N | Tremblay and Anderson 1984; EOL 2016; WoRMS 2016 |
| Peltidae |  |  |  |  | U |  |
| *Pseudcalanus* sp. |  |  |  |  | U |  |
| *Pseudcalanus* spp. |  |  |  |  | U |  |
| *Pseudobradya* sp. |  |  |  |  | U |  |
| *Pseudocalanus elongatus** |  |  |  |  | E | WRIMS 2015; ARMS 2016; EOL 2016; OBIS 2016; WoRMS 2016 |
| *Pseudocalanus newmani*† |  |  |  |  | E | Stewart and Lockhart 2005; ARMS 2016; OBIS 2016 |
| *Sarsamphiascus minutus* |  |  |  | x | E | Shih et al. 1971; ARMS 2016; EOL 2016 |
| *Sarsamphiascus parvus* |  |  |  |  | N | EOL 2016; WoRMS 2016 |
| *Temora longicornis** |  |  |  |  | E | Shih et al. 1971; OBIS 2016; EOL 2016 |
| Temoridae |  |  |  |  | U |  |
| *Thalestris longimana* |  |  |  |  | N | EOL 2016; SLB 2016; WoRMS 2016 |
| *Tisbe furcata* |  |  |  |  | E | Steele 1961; Shih et al. 1971; Stewart and Lockhart 2005; OBIS 2016 |
| *Tisbe* spp. | x (y) |  | x | x | U |  |
| *Zaus* sp. |  |  | x |  | U |  |
| **Decapoda** |  |  |  |  |  |  |
| Brachyura |  |  |  |  | U |  |
| **Echinodermata** |  |  |  |  |  |  |
| Asteroidea |  |  |  |  | U |  |
| Echinoidea |  |  |  |  | U |  |
| Holothuroidea |  |  |  |  | U |  |
| Ophiuroidea |  |  |  |  | U |  |
| **Gastropoda** |  |  |  |  |  |  |
| Caenogastropoda |  |  |  |  | U |  |
| Doridina |  |  |  |  | U |  |
| Gastropoda (dextral shell) |  |  |  |  | U |  |
| Gastropoda (sinistral shell) |  |  |  |  | U |  |
| *Lacuna* sp. |  |  |  |  | U |  |
| *Lacuna vincta* |  |  |  |  | E | ARMS 2016; EOL 2016 |
| Littorinidae |  |  |  |  | U |  |
| Nudibranchia |  |  |  |  | U |  |
| Opistobranchia |  |  |  |  | U |  |
| Patellogastropoda |  |  |  |  | U |  |
| **Hirudinea** |  |  |  |  |  |  |
| Hirudinea |  |  |  |  | U |  |
| **Hydrozoa** |  |  |  |  |  |  |
| Anthomedusae |  |  |  |  | U |  |
| Calyptoblastea |  |  |  |  | U |  |
| Campanulinidae |  |  |  |  | U |  |
| *Dicoryne conferta* |  |  |  |  | N | Mendoza-Becerril and Marques 2013; ARMS 2016; EOL 2016; WoRMS 2016 |
| *Ectopleura larynx* |  |  |  |  | N | Mendoza-Becerril and Marques 2013; ARMS 2016; EOL 2016; WoRMS 2016 |
| *Ectopleura sp.* |  |  |  |  | U |  |
| *Gonothyraea loveni* |  |  |  |  | E | Stewart and Lockhart 2005; EOL 2016; OBIS 2016; WRIMS 2016 |
| *Hartlaubella gelatinosa* |  |  |  |  | E | Stewart and Lockhart 2005 |
| *Hybocodon prolifer* |  |  |  |  | E | Stewart and Lockhart 2005; ARMS 2016; Marine Species Identification Portal 2016; OBIS 2016 |
| *Obelia longissima* |  |  |  |  | E | ARMS 2016; EOL 2016; OBIS 2016 |
| *Obelia sp.* |  |  |  |  | U |  |
| Obeliinae |  | x |  |  | U |  |
| *Pachycordyle michaeli* |  |  |  |  | N | Mendoza-Becerril and Marques 2013; OBIS 2016; WoRMS 2016 |
| *Rhizogeton nudus* |  |  |  |  | E | Calder 1972; ARMS 2016; EOL 2016; WoRMS 2016 |
| **Isopoda** |  |  |  |  |  |  |
| *Ianiropsis* sp. |  |  |  |  | U |  |
| *Idotea phosphorea* |  |  |  |  | N | EOL 2016; CaRMS 2016; OBIS 2016; WoRMS 2016 |
| Idoteidae |  |  |  |  | U |  |
| Janiridae |  |  |  |  | U |  |
| **Nematoda** |  |  |  |  |  |  |
| *Adoncholaimus* sp. |  |  |  |  | U |  |
| *Anticoma* sp. |  |  |  |  | U |  |
| *Ascolaimus elongatus* |  |  |  |  | N | R. Fisher, Salem State University, personal communication 2009; EOL 2016; OBIS 2016; WoRMS 2016 |
| *Ascolaimus* sp. |  |  |  |  | N | R. Fisher, Salem State University, personal communication 2009; OBIS 2016 |
| Axonolaimidae |  |  | x |  | U |  |
| *Axonolaimus* sp. |  |  |  |  | U |  |
| *Camacolaimus* sp. |  |  |  |  | U |  |
| *Cephalanticoma* sp. |  |  |  |  | N | R. Fisher, Salem State University, personal communication, 2013; OBIS 2016 |
| *Chromadorella* sp. |  |  |  |  | N | R. Fisher, Salem State University, personal communication, 2009; EOL 2016; OBIS 2016 |
| Chromadoridae | x (y) |  | x | x | U |  |
| *Chromadorina erythrophthalma* |  | x |  |  | N | EOL 2016; OBIS 2016; WoRMS 2016 |
| *Chromadorina* sp. 1 |  | x | x |  | U |  |
| *Chromadorina* sp. 2 | x (y) |  |  |  | U |  |
| *Chromadorita* sp. |  |  |  | x | U |  |
| Comesomatidae |  |  |  |  | U |  |
| Cyatholaimidae |  |  |  |  | U |  |
| *Daptonema* sp. 1 | x (y) |  |  |  | U |  |
| *Daptonema* sp. 2 |  |  |  |  | U |  |
| *Daptonema tenuispiculum* |  |  |  |  | N | Holovachov 2014; OBIS 2016; WoRMS 2016 |
| *Desmodora* sp. |  |  |  |  | U |  |
| *Eleutherolaimus* sp. |  |  |  |  | U |  |
| Enoplidae |  |  |  |  | U |  |
| *Enoplus sp.* |  |  |  |  | U |  |
| *Geomonhystera* sp. 1 | x | x | x |  | N | R. Fisher, Salem State University, personal communication, 2009; OBIS 2016 |
| *Geomonhystera* sp. 2 |  |  |  |  | N | R. Fisher, Salem State University, personal communication, 2009; OBIS 2016 |
| *Graphonema* sp. | x (y) |  |  |  | N | R. Fisher, Salem State University, personal communication, 2009; EOL 2016 |
| *Halalaimus* sp. |  |  |  |  | U |  |
| *Innocuonema* sp. |  |  |  |  | N | R. Fisher, Salem State University, personal communication, 2009; OBIS 2016 |
| *Leptolaimus* sp. |  |  |  |  | U |  |
| *Metalinhomoeus* sp. |  |  |  |  | N | R. Fisher, Salem State University, personal communication, 2009; ARMS 2016; OBIS 2016 |
| Monhysteridae |  |  |  |  | U |  |
| *Neochromadora* sp. 1 |  |  | x |  | U |  |
| *Neochromadora* sp. 2 | x (y) |  |  |  | U |  |
| *Onchium* sp. |  |  |  |  | U |  |
| *Oncholaimellus* sp. |  |  |  |  | U |  |
| *Oncholaimus oxyuris* |  |  |  |  | N | R. Fisher, Salem State University, personal communication, 2009; OBIS 2016; WoRMS 2016 |
| *Oncholaimus* sp. |  |  |  |  | U |  |
| *Oxystomina* sp. |  |  |  |  | U |  |
| *Paracanthonchus macrodon* |  |  |  |  | N | ARMS 2016; EOL 2016; WoRMS 2016 |
| *Paracanthonchus* sp. |  |  |  |  | U |  |
| *Prochromadora* sp. 1 |  |  |  |  | N | R. Fisher, Salem State University, personal communication, 2009; OBIS 2016 |
| *Prochromadora* sp. 2 |  |  |  |  | N | R. Fisher, Salem State University, personal communication, 2009; OBIS 2016 |
| *Prochromadora* sp. 3 | x | x |  |  | N | R. Fisher, Salem State University, personal communication, 2009; OBIS 2016 |
| *Prochromadora* sp. 4 |  |  |  |  | N | R. Fisher, Salem State University, personal communication, 2009; OBIS 2016 |
| *Prochromadorella* sp. |  |  |  |  | N | R. Fisher, Salem State University, personal communication, 2009; EOL 2016 |
| *Sabatieria* sp. |  |  |  |  | U |  |
| Selachinematidae |  |  |  |  | U |  |
| *Spilophorella* sp. |  |  |  |  | U |  |
| *Steineridora* sp. |  |  |  |  | U |  |
| *Theristus acer* |  |  |  |  | N | ARMS 2016; OBIS 2016 |
| *Tripyloides* sp. |  |  |  |  | U |  |
| *Viscosia* sp. |  |  |  |  | U |  |
| Xyalidae |  |  |  |  | U |  |
| **Nemertea** |  |  |  |  |  |  |
| Nemertea |  |  |  |  | U |  |
| **Oligochaeta** |  |  |  |  |  |  |
| Enchytraeidae |  |  |  |  | U |  |
| Naididae |  |  |  |  | U |  |
| **Ostracoda** |  |  |  |  |  |  |
| Cytherocopina |  |  |  |  | U |  |
| **Platyhelminth** |  |  |  |  |  |  |
| *Stylochus ellipticus* |  |  |  |  | N | EOL 2016; OBIS 2016; WoRMS 2016 |
| **Polychaeta** |  |  |  |  |  |  |
| Aciculata |  |  |  |  | U |  |
| Autolytinae |  |  |  |  | U |  |
| *Autolytus* sp. |  |  |  |  | U |  |
| Cirratulidae |  |  |  |  | U |  |
| *Eulalia viridis* |  |  |  |  | N | ARMS 2016; EOL 2016; OBIS 2016; WoRMS 2016 |
| *Eunoe oerstedi* |  |  |  |  | E | ARMS 2016; EOL 2016; OBIS 2016; WoRMS 2016 |
| *Harmothoe imbricata* |  |  |  |  | E | Atkinson and Wacasey 1989a,b; Stewart and Lockhart 2005; Goldsmit et al. 2014; EOL 2016; OBIS 2016; WoRMS 2016 |
| *Harmothoe* sp. |  |  |  |  | U |  |
| Lepidonotinae |  |  |  |  | U |  |
| Nephtyidae |  |  |  |  | U |  |
| Nereididae |  |  |  |  | U |  |
| *Nereis* sp. |  |  |  |  | U |  |
| *Parasabella microphthalma* |  |  |  |  | N | EOL 2016; OBIS 2016; WoRMS 2016 |
| *Pholoe* sp. |  |  |  |  | U |  |
| *Phyllodoce maculata* |  |  |  |  | E | Goldsmit et al. 2014; ARMS 2016; Marine Species Identification Portal 2016; WoRMS |
| *Phyllodoce* sp. |  |  |  | x | U |  |
| Phyllodocidae |  |  |  | x | U |  |
| Polychaeta |  |  |  | x | U |  |
| *Polydora aggregata* | x (y) |  |  |  | E | OBIS 2016; WoRMS 2016 |
| *Polydora cornuta* |  |  |  |  | N | Molnar et al. 2008; EOL 2016; WoRMS 2016; WRIMS 2016 |
| *Polydora* sp. 1 | x (y) |  |  |  | U |  |
| *Polydora* sp. 2 |  | x |  |  | U |  |
| *Polydora* sp. 3 |  |  |  |  | U |  |
| *Polydora* sp. 4 |  |  |  |  | U |  |
| Polydorine |  |  |  |  | U |  |
| Polynoidae |  |  |  |  | U |  |
| *Proceraea* sp. |  |  |  |  | U |  |
| Sabellinae |  |  |  |  | U |  |
| Serpulidae |  |  |  |  | U |  |
| Spionidae |  |  |  |  | U |  |
| **Tunicata** |  |  |  |  |  |  |
| *Ascidia* sp. |  |  |  |  | U |  |
| Ascidiacea |  |  |  |  | U |  |
| *Botryllus schlosseri* |  |  |  |  | N | Molnar et al. 2008; Ma 2012; EOL 2016; Fofonoff et al. 2016; WoRMS 2016; WRIMS 2016 |
| *Ciona intestinalis* |  |  |  |  | E | Stewart and Lockhart 2005; Ma 2012; ARMS 2016; OBIS 2016; WoRMS 2016 |
| Mogulidae |  |  |  |  | U |  |
| *Molgula manhattensis* |  |  |  |  | N | Molnar et al. 2008; Ma 2012; ARMS 2015; Fofonoff et al. 2016; WoRMS 2016; WRIMS 2016 |

**References**

AlgaeBase (2016) AlgaeBase. http://www.algaebase.org. Accessed 5 September 2016

ARMS (2016) The Arctic Register of Marine Species. http://www.marinespecies.org/arms. Accessed 19 November 2015

Atkinson EG, Wacasey JW (1989a) Benthic invertebrates collected from Hudson Strait, Foxe Channel and Foxe Basin, Canada, 1949 to 1970. Can Data Rep Fish Aquat Sci 746, Fisheries and Oceans Canada, Ste. Anne de Bellevue

Atkinson EG, Wacasey JW (1989b) Benthic invertebrates collected from Hudson Bay, Canada, 1953 to 1965. Canadian Data Report of Fisheries and Aquatic Sciences 744, Fisheries and Oceans Canada, Ste. Anne de Bellevue

Beaugrand G, Lindley JA, Helaouet P, Bonnet D (2007) Macroecological study of *Centropages typicus* in the North Atlantic Ocean. Prog Oceanogr 72:259-273. doi:10.1016/j.pocean.2007.01.002

Bousfield EL (1973) Shallow-water Gammaridean Amphipoda of New England. Cornell University Press, Ithaca

Brunel P, Bosse L, Lamarche G (1998) Catalogue of the marine invertebrates of the estuary and Gulf of St. Lawrence. Can Spec Pub Fish Aquat Sci 126, Fisheries and Oceans Canada, Ottawa

Calder DR (1972) Some Athecate hydroids from the shelf water of northern Canada. J Fish Res Board Can 29:217-228. doi:10.1139/f72-040

Carlton JT, Newman WA, Pitombo FB (2011) Barnacle invasions: introduced, cryptogenic, and range expanding Cirripedia of North and South America. In Galil BS, Clark PF, Carlton JT (eds) In the wrong place – Alien marine crustaceans: distribution, biology and impact, Springer, New York, pp 159-213

CaRMS (2016) Canadian Register of Marine Species. http://marinespecies.org/carms/index.php. Accessed 14 September 2016

Cordell JR (2001) Motile Crustacea on fouling plates. In Hines AH, Ruiz GM (eds) Marine invasive species and biodiversity of south central Alaska, Smithsonian Environmental Research Center, Maryland, pp 15-18

DAISIE (2016) Delivering alien invasive species inventories for Europe. http://www.europe-aliens.org/default.do. Accessed 14 September 2016

de Sourza Santos, Castel (2013) Comparison of four methods to estimate meiobenthic copepod *Amonardia normani* ingestion rates. Mar Biol 160:1395-2404. doi:10.1007/s00227-013-2234-4

Durbin A, Hebert PDN, Cristescu MEA (2008) Comparative phylogeography of marine cladocerans. Mar Biol 155:1-10. doi:10.1007/s00227-008-0996-x

EOL (2016) Encyclopedia of Life. http://www.eol.org. Accessed 5 September 2016

Fofonoff PW, Ruiz GM, Steves B, Carlton JT (2016) National exotic marine and estuarine species information system. http://invasions.si.edu/nemesis/ Accessed 5 September 2016

Foster JM, Thoma BP, Heard RW (2004) Range extensions and review of the caprellid amphipods (Crustacea: Amphipoda: Caprellidae) from the shallow, coastal waters from the Suwannee River, Florida, to Port Aransas, Texas, with an illustrated key. Gulf Caribb Res 16:161-175. doi:10.18785/gcr.1602.04

GBIF (2016) Global Biodiversity Information Facility. http://www.gbif.org Accessed 14 September 2016

Goldsmit J, Howland KL, Archambault P (2014) Establishing a baseline study for early detection of non-indigenous species in ports of the Canadian Arctic. Aquat Invasions 9:327-342. doi:http://dx.doi.org/10.3391/ai.2014.9.3.08

Grainger EH (1963) Copepods in the genus *Calanus* as indicators of eastern Canadian waters. In Dunbar MJ (ed) Marine distributions. The University of Toronto Press, Toronto, pp 68-94

Grainger EH (1968) Invertebrate animals. In Beals CS, Shenstone DA (eds) Science, History and Hudson Bay volume 1. Department of Energy, Mines and Resources, Ottawa, pp 351-360

Holovachov O (2014) Nematodes from terrestrial and freshwater habitats in the Arctic. Biodivers Data J 2:e1165. doi:10.3897/BDJ.2.e1165

Jeffery NW, Elías-Gutiérrez M, Adamowicz SJ (2011) Species diversity and phylogeographical affinities of the Branchiopoda (Crustacea) of Churchill, Manitoba, Canada. PLoS ONE 6:e18364. doi:10.1371/journal.pone.0018364

Johnson SC, Scheibling (1984) Reproductive patterns of harpacticoid copepods on intertidal macroalgae (*Ascophyllum nodosum* and *Fucus vesiculosus*) in Nova Scotia, Canada 65:129-141. doi: 10.1139/z87-019

Ma K (2012) Population dynamics of a non-indigenous colonial ascidian tunicate in a subarctic harbour. Thesis, Memorial University of Newfoundland

Marine Species Identification Portal (2016) Marine Species Identification Portal. http://species-identification.org/index.php Accessed 3 September 2016

Mendoza-Becerril MA, Marques AC (2013) Synopsis on the knowledge and distribution of the family Bougainvilliidae (Hydrozoa, Hydroidolina). Lat Am J Aquat Res 41:908-924. doi:103856/vol41-issue5-fulltext-11

Molnar JL, Gamboa RL, Revenga C, Spalding MD (2008) Assessing the global threat of invasive species to marine biodiversity. Front Ecol Environ 6:485-492. doi:10.1890/070064

Neves CS, da Rocha RM (2008) Introduced and cryptogenic species and their management in Paranaguá Bay, Brazil. Braz Arch Biol Technol 51:623-633. doi:10.1590/S1516-89132008000300025

Newman WW, Ross A (1976) Revision of the balanomorph barnacles; including a catalog of the species. Mem San Diego Soc Nat Hist 9:1-108

OBIS (2016) Ocean Biogeographic Information System. http://www.iobis.org. Accessed 5 September 2016

Okolodkov YB (1999) Species range types of recent marine dinoflagellates recorded from the Arctic. Grana 38:162-169. doi: 10.1080/00173139908559224

Ólafsson E, Ingólfsson A, Steinarsdóttir MB (2001) Harpacticoid copepod communities of floating seaweed: controlling factors and implications for dispersal. Hydrobiologia 453/454:189-200. doi: 10.1007/0-306-47537-5_17

Pilgrim EM, Darling JA (2010) Genetic diversity in two introduced biofouling amphipods (*Ampithoe valida* & *Jassa marmorata*) along the Pacific North American coast: investigation into molecular identification and cryptic diversity. Divers Distrib 16:827-839. doi: 10.1111/j.1472-4642.2010.00681.x

Rhodes A (2003) Methods for high density batch culture of *Nitokra lacustris*, a marine harpacticoid copepod. In: Browman HI, Skiftesvik AB (eds) The Big Fish Bang. Institute of Marine Research. Bergen, Norway, pp 449-465

Roff JC, Legendre L (1986) Physio-chemical and biological oceanography of Hudson Bay. In Martini IP (ed) Canadian Inland Seas, Elsevier, New York, pp 265-292

SeaLifeBase (2016) SeaLifeBase. http://www.sealifebase.ca/ Accessed 5 September 2016

Shih CT, Figueira AJG, Grainger EH (1971). A synopsis of Canadian marine zooplankton. Fisheries Research Board, Ottawa

Shoemaker C (1920) Report of the Canadian Arctic Expedition 1913-18, Vol. VII. King’s Printer Ottawa

Steele D (1961) Studies in the marine Amphipoda of eastern and northeastern Canada. Dissertation. McGill University

Stewart DB, Howland KL (2009) An ecological and oceanographical assessment of the alternate ballast water exchange zone in the Hudson Strait Region. Can Sci Advi Sec Res Doc 2009/008. Fisheries and Oceans Canada, Winnipeg

Stewart DB, Lockhart WL (2005) An overview of the Hudson Bay marine ecosystem. Can Tech Rep Fish Aquat Sci 2586, Fisheries and Oceans Canada, Winnipeg

Tremblay HJ, Anderson JT (1984) Annotated species list of marine planktonic copepods occurring on the shelf and upper slope of the northwest Atlantic (Gulf of Maine to Ungava Bay). Can Spec Pub Fish Aquat Sci 69, Fisheries and Oceans Canada, Ottawa

Veit-Köhler G, Fuentes V (2007) A new pelagic *Alteutha* (Copepoda: Harpacticoida) from Potter Cove, King George Island, Antarctica—description, ecology and information on its year round distribution. Hydrobiologia 583:141-163. doi:10.1007/s10750-006-0482-8

Willey A (1931) Biological and oceanographic conditions in Hudson Bay. 4. Hudson Bay copepod plankton. Contrib Can Biol Fish 6:483-493

WoRMS (2016) World Register of Marine Species. www.marinepseices.org. Accessed 5 September 2016

WRIMS (2016) World Register of Introduced Marine Species. http://www.marinespecies.org/introduced/index.php. Accessed 5 September 2016

Yorke AF, Metaxas A (2011) Interactions between an invasive and a native bryozoan (*Membranipora membranacea* and *Electra pilosa*) species on kelp and *Fucus* substrates in Nova Scotia, Canada. Mar Biol 158:2299-2311. doi:10.1007/s00227-011-1734-3
